# Using Antigen Expression of Leukemic Cells for a Fast Screening of Acute Promyelocytic Leukemia by Flow Cytometry

**DOI:** 10.3390/diagnostics11111988

**Published:** 2021-10-26

**Authors:** Vitória Ceni-Silva, Kátia Pagnano, Gislaine Duarte, Marina Pellegrini, Bruno Duarte, Konradin Metze, Irene Lorand-Metze

**Affiliations:** 1 Hematology and Hemotherapy Center, University of Campinas, Campinas 13083-878, Brazil; vitoriacenisilva@gmail.com (V.C.-S.); kborgia@unicamp.br (K.P.); gborba@unicamp.br (G.D.); marinadp@unicamp.br (M.P.); bklduarte@gmail.com (B.D.); 2 Department of Pathology, Faculty of Medical Sciences, University of Campinas, Campinas 13083-970, Brazil; kmetze@fcm.unicamp.br

**Keywords:** acute promyelocytic leukemia, diagnosis, flow cytometry

## Abstract

(1) Background: Acute promyelocytic leukemia is curable, but bleeding complications still provoke a high early mortality. Therefore, a fast diagnosis is needed for timely starting treatment. We developed a diagnostic algorithm using flow cytometric features for discrimination between acute promyelocytic leukemia (APL) and other types of acute myeloid leukemias (AML). (2) Methods: we analyzed newly diagnosed AMLs where immunophenotyping was performed at diagnosis by an 8-color protocol. The mean fluorescence intensity (MFI) of each antigen used was assessed, and those best separating APL from other types of AML were obtained by a discriminant analysis. Phenotypic characteristics of myeloblasts of normal bone marrow were used as controls. (3) Results: 24 cases of APL and 56 cases of other primary AMLs entered the study. Among non-APL AMLs, 4 had fms-related tyrosine kinase 3 gene internal tandem duplications (FLT3-ITD) mutation, 2 had nucleophosmin (NPM1) and 10 had both mutations. SSC (*p* < 0.0001), HLA-DR (*p* < 0.0001), CD13 (*p* = 0.001), CD64 (*p* = 0.004) and CD33 (*p* = 0.002) were differentially expressed, but this was not the case for CD34 (50% of non-APLs had a low expression). In the discriminant analysis, the best differentiation was achieved with SSC and HLA-DR discriminating 91.25% of the patients. (4) Conclusion: MFC could differentiate APL from non-APL AML in the majority of the cases.

## 1. Introduction

The diagnostic work-up of acute myeloid leukemia (AML) is an integrated process using cytology, immunophenotyping, cytogenetics as well as the search for specific mutations, as several subtypes are defined by cytogenetic and molecular features in the WHO 2016 classification [1,2,3]. This is especially true for acute promyelocytic leukemia (APL), a subtype of AML defined genetically by the translocation t(15;17) and the promyelocytic leukemia–retinoic acid receptor α (PML-RARA). APL is highly curable if appropriate therapy based on all-trans retinoic acid (ATRA) is timely started, preventing bleeding due to a consumption coagulopathy, which is responsible for a high early mortality rate [4,5]. Therefore, there is a need for a rapid diagnostic screening. Results of cytogenetics and molecular tests are not readily available and fluorescence in situ hybridization (FISH) is not available in many laboratories. Therefore, most of the times, the first screening is made by cytology and immunophenotyping in many institutions, since the results of cytogenetics and molecular tests are not immediately available.

Several works have reported that leukemic cells in APL usually do not express CD34 and HLA-DR, but this is also the case in several other types of AML, mainly those presenting the NPM1 mutation [2,6,7,8]. Recently, some groups [9,10] have described a method using multidimensional analysis in radar plots of the Kaluza software, to screen APL compared to non-APL acute myeloid leukemia. However, this method is dependent on a specific software and its reproducibility requires a standardization of antibodies, fluorochromes and analysis. 

Our aim was to develop a diagnostic algorithm based on the expression intensity of several antigens examined by multiparametric flow cytometry (MFC) that depends only on internal standardization and could be performed with several antibody combinations and software of analysis, but could reliably discriminate between APL and other types of AML. 

## 2. Material and Methods

### 2.1. Study Design

We reanalyzed the flow cytometric files of all newly diagnosed cases of de novo AML entering our institution between 2017 and 2019 and where cytogenetics and molecular diagnosis for PML/RARA, nucleophosmin (NPM1), fms-related tyrosine kinase 3 gene internal tandem duplications (FLT3-ITD) and second tyrosine kinase domain (FLT3-TKD) were available. These techniques had been introduced in the routine diagnostic work-up of AML at our Institution before 2017. The diagnosis had been made by WHO 2016 criteria based on peripheral blood counts, bone marrow (BM) cytology, cytogenetics, immunophenotyping and molecular analysis. Cases of APL with variant karyotypes had been excluded.

Cytogenetics had been performed in bone marrow cells by conventional G banding. Screening for nucleophosmin (NPM1), FLT3-ITD and FLT3-TKD mutations had been performed in genomic DNA with labeled primers and examined by fragment analysis. FLT3-ITD/wt allelic burden was calculated as the ratio of the area under the curve of mutant and wild-type alleles (FLT3-ITD/FLT3wt) [11,12,13]. PML-RARA rearrangements were detected by qualitative RT-PCR [13].

The study was approved by the local Ethics Committee (proc nr. 29661720.6.0000.5404).

### 2.2. Flow Cytometric Analysis

All BM samples had been collected in EDTA and processed within 24 h after collection using an eight-color platform with at least 2 tubes (Supplementary Tables S1 and S2). At least 100,000 events had been acquired on a BD FACSCanto II equipment. Analysis was performed in the INFINICYT^TM^ 1.8 software (Cytognos SL, Salamanca, Spain). The mean fluorescence intensity (MFI) of each antigen tested was assessed for each case and compared with that of normal myeloblasts, obtained from cases where bone marrow MFC had been performed for the diagnostic work-up of peripheral blood cytopenias of unknown origin using the same flow protocol and where no aberrancies had been found. The SSC of the blasts was measured as the ratio between the value obtained for myeloid progenitors and that of the lymphocyte gate, used as internal control.

### 2.3. Statistical Analysis

Descriptive statistics was performed with non-parametric tests. In all AML cases, antigen expression was considered as positive when its MFI was above the 10% percentile of normal myeloid progenitors. The differences among groups were tested by Mann–Whitney and Kruskall–Wallis tests. All variables significantly different in the Mann–Whitney tests were analyzed in linear discriminant analyses [14,15]. Variables with a non-normal distribution entered this analysis after logarithmic transformation in order to approximate the data to a Gaussian distribution. In a first step, we ran a discriminant analysis individually. In a second step, we looked for the combination of variables that could best separate APL from non-APL patients in a multivariate discriminant analysis by testing all variables with significant differences between both groups simultaneously in a stepwise procedure. For this purpose, we used the stepwise function of the SPSS software which selects the best variables for an optimized discriminant model in a stepwise manner. The intrinsic stability was evaluated by the jackknife (leave-one-out) method [14]. Winstat and SPSS 15 softwares were used for the calculations.

## 3. Results

Only 24 patients with APL and 56 with other AML subtypes fulfilled the inclusion criteria. Regarding the APL patients, the final diagnosis was made by both cytogenetics and molecular biology in 11 cases and only by molecular testing in 12. In only one patient, the diagnosis was based only on the karyotype. The demographic characteristics of the patients are shown in Table 1. As expected, APL patients were significantly younger and had significantly lower peripheral leukocyte counts than non-APL cases. Among these last, the majority was of the intermediate risk group according to the ELN criteria.

Regarding phenotypic features (Table 2), 17 of 24 (71%) cases of APL had an increased SSC compared to normal myeloblasts and all were negative for HLA-DR and CD34 by this criterium. APL had an increased expression of CD13 in 23 patients compared with normal myeloid progenitors (Supplementary Figure S1).

Comparing APL with non-APL cases, expression of CD13, CD64 and CD33 was higher in the former, while that of CD117 showed similar values. CD34 and HLA-DR were expressed in only 18 (32%) and 25 (44%), respectively, of non-APL cases, and there was a positive correlation between expression of CD34 and HLA-DR (0.385; *p*: 0.007).

**Figure 1:**
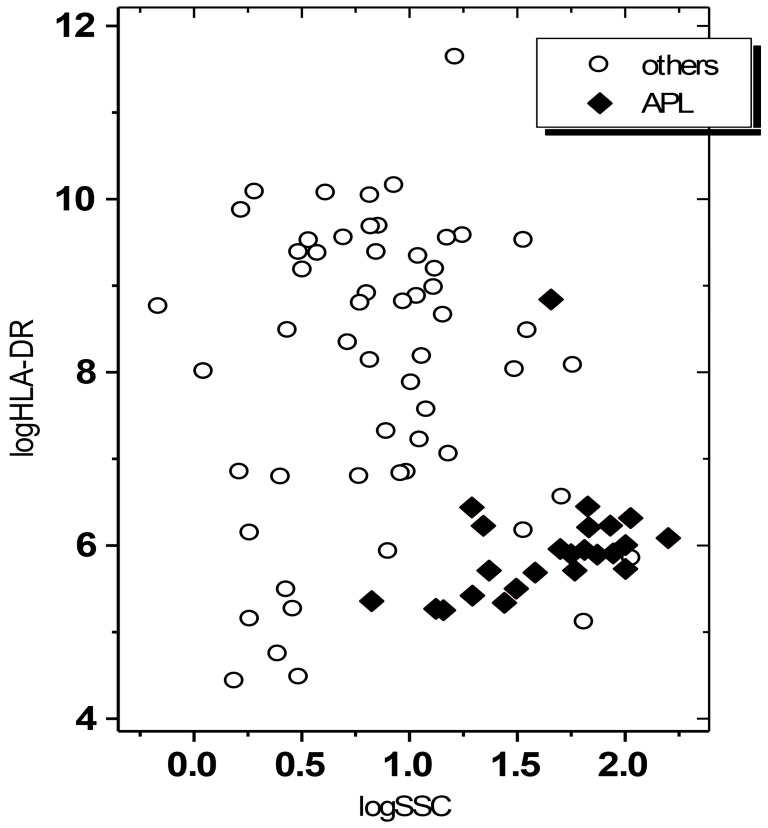
Graphical representation of the distribution of the values of “SSC” and “HLA-DR” that composed the best model: there is little overlap between APL and the non-APL cases.

The results of the discriminant analysis are presented in Table 3. In the multivariate analysis, the software suggested the “ratio SSC blast/SSC lymphocyte” and “MFI of HLA-DR” as the best model, which could discriminate APL from non-APL in 91.25% of cases and in 90% after the leave-one-out method (Figure 1). If “SSC” was not included in the analysis, a model formed by HLA-DR, CD13 and CD64 was obtained, which discriminated 86.1% of the cases and 84.8% cases after the leave-one-out method. The only model containing CD34 was obtained if this marker was examined together with only CD13, discriminating 69.6% of the cases and 69.6% after the leave-one-out method.

Among the patients with APL, five presented the FLT3-ITD mutation and four FLT3-TKD. Both mutations were found in two patients. NPM1 mutation was not found in any of the cases.

The karyotype of non-APL cases could be retrieved in 39/56 cases. Among them, 15 had a normal karyotype, while t(8;21) was found in four, del(5) was found in four, +8 in two, alterations in chromosome 17 in two and several other abnormalities in 12 patients. 

Mutation analysis could be retrieved in 48/56 of non-APL cases. In 30 patients, no mutation was found. One was found in seven cases: FLT3-ITD in four cases, NPM1 in two and FLT3-TKD in one patient. Eleven cases presented two mutations: FLT3-ITD and NPM1 were found in 10 cases and FLT3-TKD and NPM1 were seen in one case each. Patients without mutations had lower peripheral leukocyte counts and showed a higher expression of CD34 (*p* < 0.0001) and HLA-DR (*p* = 0.008).

## 4. Discussion

A rapid diagnostic screening for APL is essential for timely starting an appropriate treatment, thus preventing complications due to bleeding. This screening is usually done by cytology and immunophenotyping, or by immunocytochemistry with anti-PML staining [16] or FISH [17], which depends on the availability of the appropriate reagents.

Recently, Kárai et al. [9] described a screening for APL using the multiparametric radar plot analysis of the Kaluza software for immunophenotyping. In this technique, a radar plot was constructed based on three different types of AMLs, including one FAB-M2, FAB-M4 and APL case, and searching for the parameters which best separated the three types of blasts. However, the authors admit that this technology would need a good standardization and the same antibodies and fluorochromes in all laboratories. Gupta et al. [10] confirmed these results by comparing APL and NPM1^+^ AML.

We aimed to develop a diagnostic algorithm based on the intensity of antigen expression and comparing the expression with that of normal myeloid progenitors processed with the same equipment and antibody combinations. Positivity for each marker was defined as above the 10% percentile of the values found for the controls. This approach is feasible in every laboratory, assuming that an internal standardization of instrument settings and antibody combination is made. 

We could detect several antigens differentially expressed in APL, such as CD13, CD35, CD64 and HLA-DR. The expression of CD13 and CD64 was highly variable among all cases in both groups but tended to be higher in APL, as also described by Liu at al [18]. Expression of CD35 was low in all cases. HLA-DR was negative in all our APL cases, but it was positive in only 44% of the non-APL cases. Concerning CD34, all our cases of APL showed a lower expression compared to that of normal myeloid progenitors. Among non-APL cases, expression of this antigen was also decreased compared to normal blasts in 67%. Interestingly, the CD34+ AMLs had a lower peripheral leukocyte count and showed no NPM1 and FLT3 mutation. The ratio “SSC blasts/SSC lymphocytes” was increased in 71% of APL cases, but only in 10% of non-APL cases. Although it has been often reported that SSC is increased in APL, this feature has not been highlighted as a discriminant between this type and other forms of AML [19].

The best model in the discriminant analysis was based on the ratio between SSC blasts/SSC lymphocytes (high in APL) and a low MFI of HLA-DR, which separated APL from non-APL AML in most cases. MFI of CD34 entered in only one model tested.

Choosing a cut-point is often somehow arbitrary. Moreover, dichotomizing data initially collected as continuous data (positive or negative for an antigen expression) causes an important loss of information, reduces statistical test power, may increase diagnostic uncertainty, introduce bias and lead to oversimplification of the results [20]. We overcame this problem by analyzing the MFI values, which allows us to extract more valuable information from the expression of the antigens assessed. Thus, expression of HLA-DR alone was able to correctly discriminate 77.5% of the patients. The MFI of CD34 was not useful for this screening, as 67% of the non-APL AMLs also had a low expression of this marker. The introduction of the variable “SSC”, which represents an indirect measure of the cytoplasmic characteristics, increased the screening potential to higher than 90%. The SSC ratio alone could correctly discriminate 85.2% of the patients. By combining SSC with MFI HLA-DR in the discriminant analysis, we achieved a high discrimination rate (91.25%). Since this value dropped only slightly after the leave-one out test, the discriminant analysis showed a good intrinsic stability of the mathematical model. We did not examine any microgranular/hypogranular variant of APL, but our approach could also be suitable to detect this form, as a model not considering SSC was able to discriminate 86.1% of the cases. This must be proven analyzing real cases.

In contrast to the work of Karai et al. [9], we included 80 patients in the study, thus representing better the dispersion of the variables in the population of AML subtypes. In contrast to other methods, our technique applies variables which can be achieved by different flow cytometric protocols. In summary, our technique permits a simple, rapid and efficient screening of APL from other types of AML based on two standardized flow cytometric parameters.

## Figures and Tables

**Table 1:** Demographic characteristics of the patients. Median (percentiles 10–90%).

	APL *n* = 24	Other AMLs *n* = 56	*p* Value
Age	39.5 (23–56)	62 (26–81)	<0.0001
Male/female	8/16	30/26	
Hemoglobin g/dL	7.8 (5.7–10.5)	7.9 (6.4–10.5)	0.46
Leukocytes × 10^9^/L	6.39 (0.6–53.4)	13.76 (10.7–144.3)	0.036
Platelets × 10^9^/L	27 (7–47)	36 (14–24.98)	0.023
ELN risk category *			
Favorable		14	
intermediate		26	
High risk		9	

* Among 49 cases.

**Table 2:** Side scatter (SSC) and mean fluorescence intensity (MFI) of the antigens tested. Median (percentiles 10–90%). Comparison by the Kruskal–Wallis test.

	Controls *n* = 10	APL *n* = 24	Non-APL *n* = 56	*p*
SSC *	2.12 (1.4–4.6)	5.6 (3.1–7.5)	2.3 (1.3–4.6)	<0.0001
MFI CD45	2409 (1194–3147)	2825 (1848–4801)	3679 (2139–6521)	0.02
MFI CD13	1094 (449–2731)	5497 (1508–13,069)	2055 (401–7357)	0.001
MFI CD35		600 (223–1169)	209 (100–831)	<0.0001
MFI CD64		1313 (392–4698)	381 (51–3674)	0.004
MFI CD33 **		10,505 (9766–13,563)	2961 (903–12,196)	0.002
MFI HLA-DR	8974 (5910–11,226)	366 (201–629)	4495 (187–20,366)	<0.0001
MFI CD34	2970 (2711–9214)	678 (345–1794)	1104 (103–9383)	0.25
MFI CD117	2611 (981–6953)	2100 (661–5032)	2426 (502–8357)	0.45

* Ratio SSC blasts/SSC lymphocytes; ** Analyzed in 7 cases of APL and 37 cases of other AMLs.

**Table 3:** Univariate discriminant analyses for the antigens that showed significantly different expressions in Table 2.

Variable	Cases Correctly Classified
SSC	85.2%
CD13	63.3%
CD64	63.2%
HLA-DR	77.5%
CD34	60.0%

## Data Availability

Data available with authors on demand.

## Supplementary Materials

The following are available online at https://www.mdpi.com/article/10.3390/diagnostics11111988/s1, Figure S1: Dot plots comparing the flow features discriminating APLand AML with a normal karyotype and NPM1 mutation., Table S1: Antibody combinations used. Table S2: Antibody clones.
