# Supplementary material for: Using Antigen Expression of Leukemic Cells for a Fast Screening of Acute Promyelocytic Leukemia by Flow Cytometry
Source: Diagnostics (Basel). 2021 Oct 26;11(11):1988. doi: 10.3390/diagnostics11111988 (PMC8625952; doi:10.3390/diagnostics11111988)
Supplement: Supplementary file 1 [file diagnostics-11-01988-s001.zip › diagnostics-1312658-supplementary.pdf]

# Supplementary Materials

**Supplementary Tabel S1.** Antibody combinations used.

| | FITC | PE | PERPCy5.5 | PECy7 | APC | APCH7 | V450 | V500 |
|---|---|---|---|---|---|---|---|---|
| **1** | CD16 | CD13 | CD34 | CD117 | CD11b | CD14 | HLA-DR | CD45 |
| **2** | CD35 | CD64 | CD34 | CD14 | CD33 | CD14 | HLADR | CD45 |

**Supplementary Table S2.** Antibody clones.

| Antibody | Clone |
|---|---|
| CD16 | CLB/FcGran1 |
| CD35 | E11 |
| CD13 | WM15 |
| CD64 | 10.1 |
| CD34 | 8G12 |
| CD117 | 104D2 |
| CD11b | ICRF44 |
| CD33 | WM53 |
| CD14 | MφP9 |
| HLA-DR | G46-6 |
| CD45 | HI30 |

All antibodies were purchased from Becton Dickinson (BD).

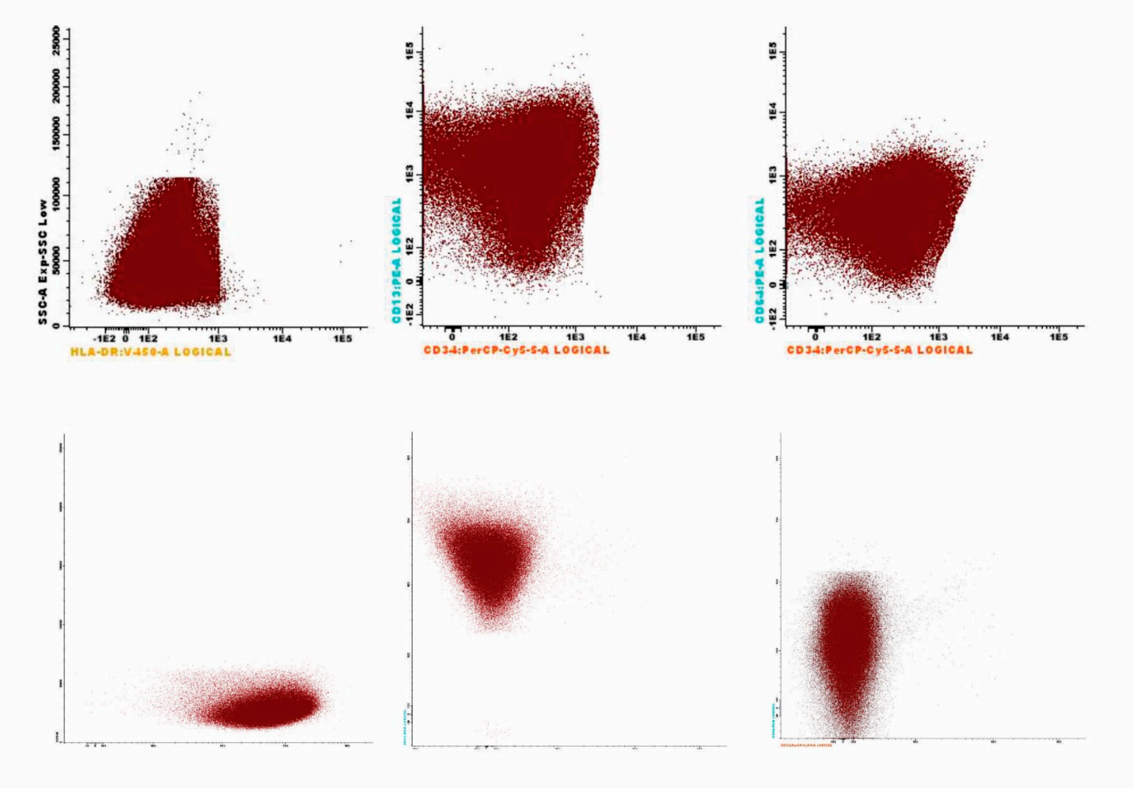


**Supplementary Figure S1.** Dot plots comparing the flow features discriminating APLand AML with a normal karyotype and NPM1 mutation. APL had a higher SSC, was negative for HLA-DR and CD34, and had a higher expression of CD13 and CD64.
